# Close and regular surveillance is key to prevent severe complications for Peutz-Jeghers syndrome patients without gastrointestinal polyps: case report of a novel *STK11* mutation (c.471_472delCT) in a Chinese girl

**DOI:** 10.1186/s12893-018-0357-8

**Published:** 2018-04-23

**Authors:** Zi-Ye Zhao, Yu-Liang Jiang, Bai-Rong Li, Jing Li, Xiao-Wei Jin, En-Da Yu, Shou-Bin Ning

**Affiliations:** 1 Department of Colorectal Surgery, Changhai Hospital, 168 Changhai Rd, Shanghai, 200433 China; 2 Department of Gastroenterology, Airforce General Hospital of PLA, 30 Fucheng Rd, Beijing, 100142 China

**Keywords:** Peutz-Jeghers syndrome, *STK11* gene, Hamartoma, Polyposis, Enteroscopy

## Abstract

**Background:**

Peutz-Jeghers syndrome (PJS) is a Mendelian disease characterized by gastrointestinal hamartomas, mucocutaneous pigmentation (MP), and increased cancer risk. Serine/threonine kinase 11 (*STK11*) is the only validated causative gene in PJS. Clinical observation reveals MP and intussusception in childhood are more frequent and severe than in adults.

**Case presentation:**

We report here a girl without a positive family history, who grew oral and fingertip MP at her age of 2 and got abdomen dull pain from 7 years old. Endoscopy revealed no obvious polyps in the stomach or the colon until 10 years old, when she received enteroscopy. Tens of polyps were resected during enteroscopy, and pathological examination confirmed them hamartomas. A heterozygous deletion in *STK11*, c.471_472delCT, was detected in the proband but not in her parents, which is not recorded in databases.

**Conclusion:**

The mutation we reported here is a novel one and a de-novo one, so our results enlarge the spectrum of STK11. We speculate close and regular endoscopy especially enteroscopy is necessary for complication prevention when the former endoscopy discovers no polyps temporarily in a child of suspect PJS.

## Background

Peutz-Jeghers Syndrome (PJS, OMIM 175200) is an autosomal dominant disorder characterized by gastrointestinal (GI) hamartomatous polyps, mucocutaneous pigmentation (MP) and an increased risk for the development of GI and various extra-GI malignancies [1]. Germline mutations in serine/threonine kinase 11 (*STK11*, OMIM 602216) gene which impairs its kinase activity are considered to cause PJS, and more than 400 of them identified in patients with PJS have been recorded in Human Gene Mutation Database (HGMD, http://www.hgmd.cf.ac.uk).

Among reported cases of PJS, more than 30% of them develop polyp-related symptoms by age 10 years and 50% by 20 years [2]. In children with PJS, intussusception due to GI polyps is the most important symptom, while PJS-associated tumors are primary in adults [3]. van Lier et al.[4] reported cumulative intussusception risk in PJS of 15% by age 10 years and 50% by age 20. Because of this background, it is important to make a set of strategies for screening and surveillance in young PJS patients, especially when family history is absent.

Here, we report a 11 year-old PJS girl without a positive family history characterized by MP and GI polyps, and Sanger sequencing confirmed a novel mutation in *STK11* (c.471_472delCT) as the causative one.

## Case presentation

### Clinical information

**Fig. 1:**
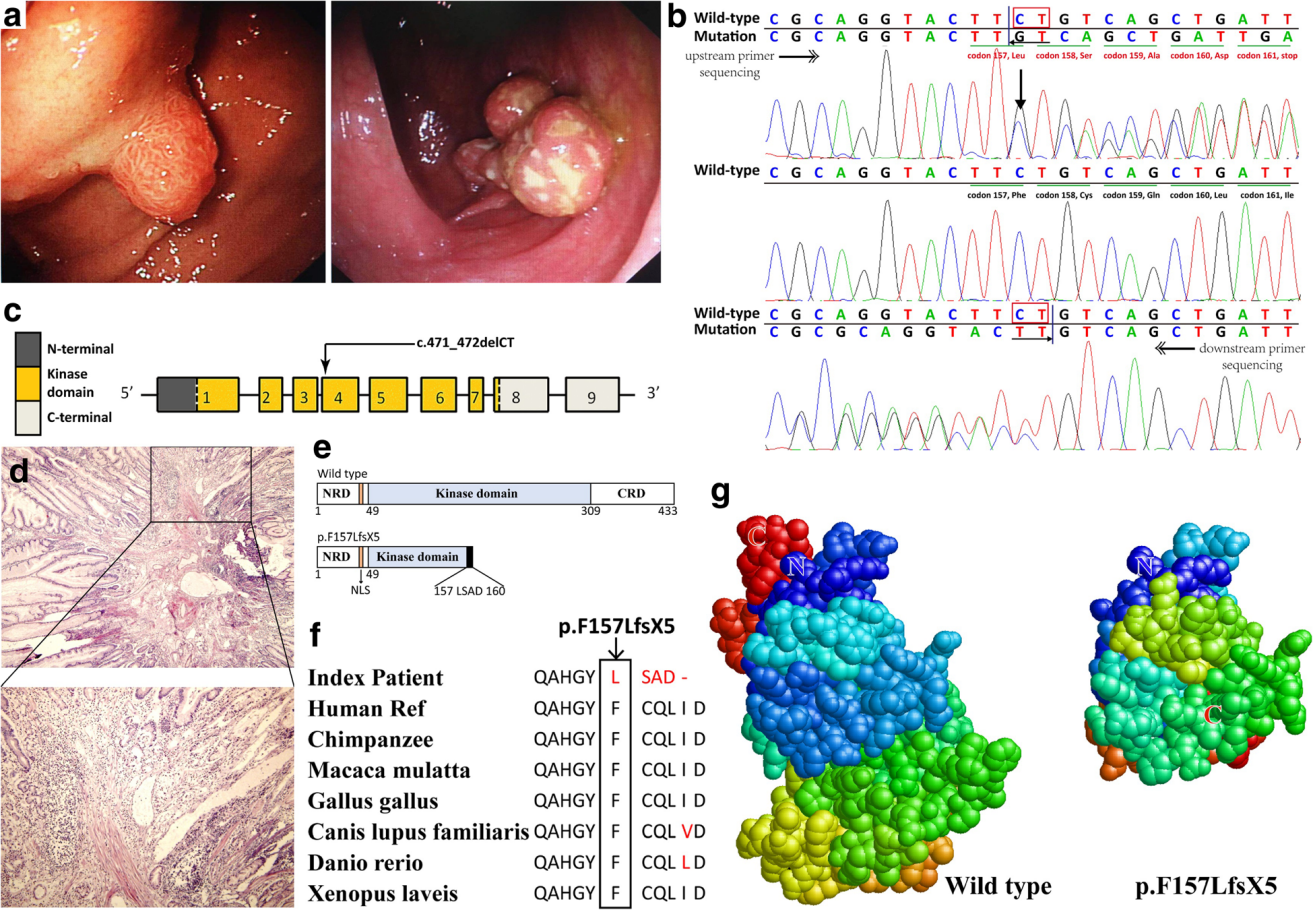
Endoscopic findings, pathology, electropherogram and mutant protein structure of the index patient. **a**. Endoscopic view of the largest polyp within the stomach (Left) and the colon (Right). **b**. Sanger sequencing forward and backward revealed a heterozygous deletion, c.471_472delCT. **c**. The structure of *STK11* gene. This novel mutation is within exon 4. **d**. Representative hematoxylin-eosin-stained tissue slices of the ileal polyp specimens confirms hamartomatous. Up, × 40 magnification; low, × 100 magnification. **e**. Schematics of the secondary structure or functional domains of the STK11 protein. The mutant protein (p.F157Lfs*5) results in a partial loss of kinase domain and a complete loss of the C-terminal domain compared to the wild type. NLS, Nuclear localization signal, NRD or CRD, N- or C-terminal regulatory domain. **f**. Evolutionary conservation of amino acid residues altered by c.471_472delCT (p.F157Lfs*5) across different species. G**.** Predicted by Swiss-Model online software, the mutant protein (p. F157Lfs*5) turns into an abnormal shape with loss of main functional domain compared to the wild type

The 11 year-old girl of Hui nationality (a race of Muslim in China) from East China came to Airforce General Hospital at her age of 10, who was diagnosed of suspect PJS in other hospital. Multiple MP in lips, cheeks and fingertips were observed by her families when she was 2 years old, and the occasional seizure of abdominal dull pain did not draw their attention since this family had no history of PJS. At the age of 7, the girl was brought to a major pediatric hospital, but gastroscopy and colonoscopy revealed no obvious polyps, after which the diagnosis of PJS was only suspected but not confirmed. At her age of 10, paroxysmal periumbilical cramps lasted for 2 days, which convinced the parents arranged their daughter a thorough examination. After being referred to our department, we arranged her for double-balloon enteroscopy (DBE). DBE both anterograde and retrograde revealed tens of polyps in the gastric fundus and 4 polyps in the colon, within which the biggest one was as large as 3 cm in diameter, and at the same time endoscopic polypectomy was performed (Fig. 1a). Postprocedural pathological examination confirmed them hamartomas, and the diagnosis of PJS was affirmed (Fig. 1d). The symptoms relieved largely after enteroscopy, and she lives a life uneventfully after that.

### Mutation analysis

During her hospitalization, we recruited her in our PJS surveillance and research cohort and collected the blood samples of herself and her parents. Written informed consents were obtained from her parents as the girl’s guardians. Genomic DNA extraction, polymerase chain reaction (PCR), purification and sequencing were performed essentially as previously described, mainly using animal genomic DNA kit (TSP201), gel extraction kit (GE0101), and 2 × modified DNA polymerase mix (TSE004, TsingKe Biotech, Beijing, China) [5]. The results were used to performance sequence alignment according to *STK11* gene sequence (NP_000446.1 and NM_000455.4 in GRCh38.p7).

In order to rule out polymorphisms, 100 chromosomes from 50 unrelated control individuals who came to our department for gastric polyps treatment in September, 2016 were also screened for the presence of the mutation. All biological samples were collected after written consent being acquired. This study was approved by the Medical Ethics Committee, Airforce General Hospital of PLA.

A survey of *STK11* gene revealed a heterozygous germline deletion c.471_472delCT in exon 4 in the patient’s genomic DNA (Fig. 1b and c), while it was not detected in the parents’ or the 50 unrelated control individuals’. This mutation has not been reported in literatures or recorded in mutation databases such as dbSNP, ClinVar and HGMD. It causes a translational frameshift, and a premature stop codon appears in codon 161 (p. F157Lfs*5), which results in a partial loss of kinase domain and a complete loss of the C-terminal domain compared to the wild type (Fig. 1e).

### Structure prediction of the mutant protein and analysis of evolutionary conservation of amino acid residues

The homology modeling programs, Swiss-Model online software (http://swissmodel.expasy.org), was used to develop an appropriate model to mimic the effects of the mutated region [6]. Evolutionary conservation of amino acid residues altered was analyzed by comparing across different species (https://www.ncbi.nlm.nih.gov/protein/STK11).

Predicted by Swiss-Model online software, the mutant protein turns into an abnormal shape and loses main functional domains compared to the wild type (Fig. 1g). Evolutionary conservation analysis of amino acid residues showed these impaired amino acid residues were most evolutionary conserved (Fig. 1f). Taken together, this mutation is considered as a novel “pathogenic” one in *STK11* causing PJS according to American College of Medical Genetics and Genomics (ACMG) classification system (Table 1).

**Table 1:** Classification of multiple evidences about the novel mutation

Evidences	c.471_472delCT
Population data	Absent in 50 controls and population databases (PM2)
Computational and predictive data	Predicted null variant (frameshift mutation included) in *STK11* where LOF is a mechanism of PJS (PVS1)
Functional data	Not applicable
Segregation data	Cosegregation with PJS (PP1)
De novo data	De novo (without paternity & maternity confirmed) (PM6)
Conclusion	Pathogenic (1 PVS1 and 2 PM and 1 PP)

## Discussion and conclusions

In this report, we revealed a novel mutation in *STK11* causing PJS in an 11 year-old girl without a positive family history. The mutation was not recorded in databases, and it was not detected in her patients or 50 unrelated control individuals. Furthermore, structure prediction and evolutionary conservation analysis present the pathological effect of it, we conclude that this mutation is disease-specific and not a polymorphic variant of the *STK11* gene.

PJS is named after two physicians who made great contributions to this disorder. Peutz described a family with autosomal dominant inheritance of GI polyposis and pigmented mucous membranes, and Jeghers defined the coexistence of MP and GI polyposis as a distinct clinical entity [7]. The pathogenic gene, *STK11*, was first cloned in 1997 [8], which encodes a 433-amino-acid-residue protein acting as a tumor suppressor. With the help of direct sequencing and multiplex ligation–dependent probe amplification (MLPA), the mutation detection rate of STK11 in PJS is raised to over 60% [9]. Among reported mutations, frameshift or nonsense ones are most common types. The STK11 protein is mainly comprised of an N-terminal non-catalytic domain, a catalytic kinase domain, and a C-terminal non-catalytic regulatory domain [10]. Among reported *STK11* mutations (HGMD), most variants are located in the region of catalytic kinase domain (amino acids 49–309) which leads to the loss of kinase activity [11]. In this case, the mutant protein lacks main functional domains compared to the wild type, so the novel mutation probably a pathogenic one. This mutation broadens the pathological mutation spectrum of *STK11* gene. Till now, no certain relationship between genotype and phenotype has been determined (Table 2), which should be further explored through continuous work.

**Table 2:** Information about genotype-phenotype correlation of *STK11* mutations in PJS

First author	Year	Country	Subjects^a^	Mutation detection rate	Germline mutation		Clinical risk suggestion
					Site	Type	
Wang [17]	2014	China	35	67.3%	Exon 7	–	High incidence of GI polyp dysplasia
van Lier [4]	2011	Netherlands	77	96.3%	–	–	Independent of *STK11* mutation status
Mehenni [18]	2007	Switzerland	27	28.7%	–	–	No significant influence
Mehenni [19]	2006	EU, AS	40	26.8%	Exon 6	–	Higher risk of cancer
Hearle [20]	2006	EU, AU, US	297	70.9%	–	–	No significant influence
Schumacher [21]	2005	^b^	132	–	Exon 1-7 - Exon 4-5	Deletion Splice site Missense	Rarely associated with cancer Significantly associated with cancer Associated with malignancies
Lim [22]	2004	EU, AU, US	78	32.5%	Exon 3	–	Higher cancer risk

^a^Subjects with detected *STK11* mutation. ^b^Subjects most from the literature. - No data

*EU* Europe, *AS* Asia, *AU* Australia, *US* United States

For children without a family history, PJS is hard to diagnose until they suffer from severe GI complications [12]. On this condition, an open surgery is often unpreventable. When the diagnosis of PJS is suspected in a child based on the typical MP together with uncertain GI symptoms, a screening test is preferable. According to the recommendations for management 2010, baseline colonoscopy and upper GI endoscopy should be performed at the age of 8 [2]. While Goldstein et al. [13] suggested an earlier initial screening age might be beneficial for children suspect of PJS.

The recommendation screening methods include gastroscopy and colonoscopy, but the situation here emphasizes the importance of follow-up after a negative initial endoscopy. In PJS, polyps prefer to be located in small bowel rather than in stomach or colon, so it is necessary to exam the small bowel when there is no findings in the stomach and colon. With the help of DBE, small bowel polyps can be detected and resected before they cause complications such as intussusception and obstruction due to GI polyps, and open surgeries can be largely avoided [14]. Belsha et al. [15] proved that polypectomy by DBE is effective in managing pediatric patients with PJS by a cohort of 16 patients with 6 years follow-up. Another lesson brought by this case is that PJS polyps are easy to grow in children, which justifies the close follow-up after negative endoscopy. All above, we suggest DBE is the proper choice after negative gastroscopy and colonoscopy if GI symptoms persist. Luckily, no severe GI complications happened to her before she received DBE. In our experience of managing 131 PJS patients who had abdominal surgeries for intestinal obstruction before, 86.3% (113) of them avoided a second open surgery with the help of regular follow-up by DBE [16].

## Abbreviations

ACMG: American College of Medical Genetics and Genomics
DBE: double-balloon enteroscopy
GI: gastrointestinal
HGMD: Human Gene Mutation Database
MP: mucocutaneous pigmentation
PCR: polymerase chain reaction
PJS: Peutz-Jeghers syndrome
*STK11*: Serine/threonine kinase 11
